# Pleural plasmacytomas in a patient with multiple myeloma relapse

**DOI:** 10.1016/j.rmcr.2022.101777

**Published:** 2022-11-13

**Authors:** Harith Al-Ataby, Amna Al-Tkrit, Samah Ali, Chandula Seneviratne, Mohamed Omballi

**Affiliations:** a Department of Pulmonary and Critical Care, University of Toledo, Ohio, USA; b Department of Pathology, University of Toledo, Ohio, USA

**Keywords:** Multiple myeloma relapse, Pleural plasmacytoma, Extramedullary plasmacytoma, FDG-PET/CT, Plasma cell neoplasm, Myelomatous pleural effusion, Solitary plasmacytoma of bone

## Abstract

Extramedullary plasmacytoma with pleural involvement in the setting of relapsed multiple myeloma (MM) is a rare yet serious condition, which is associated with an adverse prognosis. This report describes a patient with MM who was in complete remission but relapsed with multiple pleural plasmacytomas. The diagnosis was established in a timely manner and the patient was started on appropriate treatment.

## 1 Introduction

Extramedullary plasmacytoma may sometimes be seen as an initial manifestation or a part of relapse in patients with multiple myeloma (MM). In rare cases, extramedullary disease may involve the pleura (pleural plasmacytoma) and can result in nodular pleural thickening with or without pleural effusion. Pleural plasmacytoma can result in nodular pleural thickening with or without pleural effusion and associated with an adverse prognosis. These tumors are extremely rare and have not been widely described in the medical literature. In this report, we describe a case of multiple pleural plasmacytomas in a patient with MM relapse. The patient was diagnosed using fluorodeoxyglucose-positron emission tomography/computed tomography (FDG-PET/CT) and biopsy, and timely treatment with combination chemotherapy was initiated.

## 2 Case presentation

A 66-year-old woman with a history of multiple myeloma presented to our hospital referred from outpatient clinic with dyspnea on exertion which gradually worsening for the last one month. Physical examination was significant for decrease breathing sound and dull percussion on the left hemithorax.

The patient was initially diagnosed with immunoglobulin A (IgA) lambda multiple myeloma in 2011, when she developed back pain and was found to have a lytic lesion involving the T7 vertebra. A bone marrow biopsy revealed borderline hypocellular bone marrow (30%) with 10% plasma cells expressing CD38, CD56, and CD136 with cytoplasmic lambda light chain. The patient received palliative radiation therapy to the lower thoracic vertebrae for 14 days, and subsequently, was started on bortezomib. She received maintenance therapy with bortezomib for 7 years, after which she was taken off the drug with regular surveillance with serum protein electrophoresis (SPEP), urine protein electrophoresis (UPEP), immunofixation, serum free light chain measurement, urine kappa/lambda light chain measurement, and whole-body positron emission tomography/computed tomography (PET/CT) performed every 6 months.

The patient remained in remission for almost 9 years. However, 18 months ago, a PET/CT scan revealed a new PET-avid soft tissue mass along the posterior pleural surface of the right lower lobe with an erosion of the adjacent right ninth rib as well as the right-sided cortex and transverse process of the T9 vertebra. Bilateral PET-avid pulmonary lymphadenopathy was also present. T9 bone biopsy showed infiltration of monoclonal plasma cells and the bone marrow biopsy showed only 8% plasma cells, thus confirming the diagnosis of solitary plasmacytoma of bone (SPB) with minimal marrow involvement. Laboratory result showed normal level of free kappa light chains, free lambda light chains, with normal free kappa/lambda ratio. Local therapy was elected and palliative radiotherapy for the lower thoracic vertebrae was performed.

**Fig. 1:**
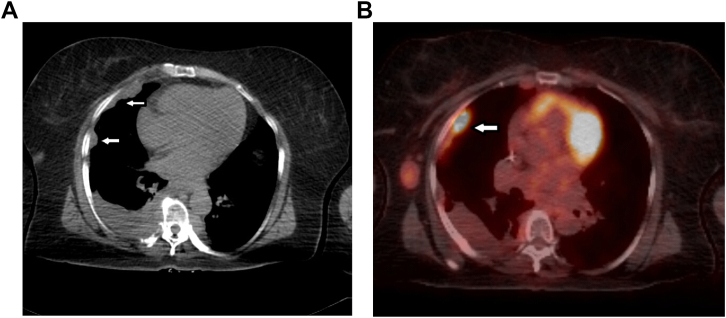
(A) Axial images chest CT scan which show soft tissue pleural based masses. (B) Axial images of PET/CT scan which show PET avid pleural based masses.

1 month ago, the patient started complaining of significant shortness of breath on exertion. A PET scan was performed and revealed multiple lytic lesions and PET-avid foci in the skeleton, including the occipital and parietal bones of the skull, left maxilla, thoracic and lumbar spine, sacrum, both femurs, sacrococcygeal joint, and the right anterior iliac crest. She was also found to have multiple pleural-based, PET-avid soft tissue lesions in both hemithoraces and a soft tissue nodule in the right lateral chest wall with moderate size right sided pleural effusion (Fig. 1). Laboratory result showed that free kappa light chains 1.38 mg/dl, free lambda light chains 12.13 mg/dl, free kappa/lambda ratio 0.11, IgG 607 mg/dl, IgA 2770 mg/dl, IgM 14 mg/dl, total protein 7.8 g/dl.

**Fig. 2:**
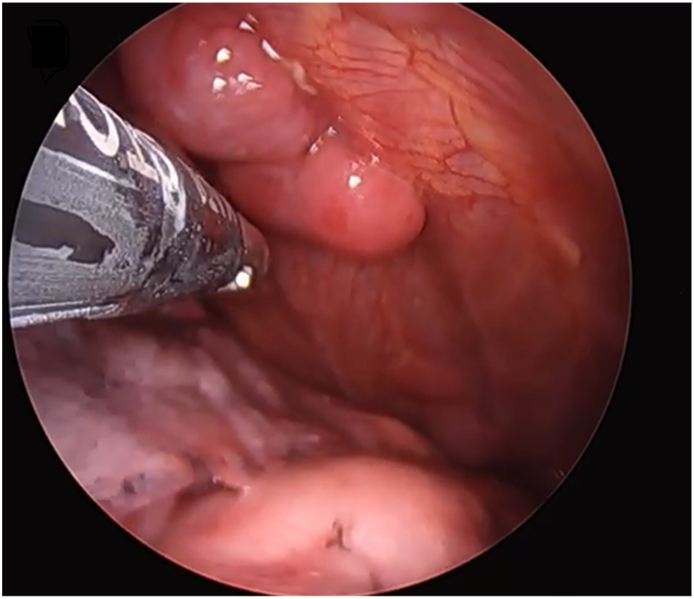
Axial images of PET/CT scan which shows significant reduction of the PET activity pleural based masses.

During the current hospitalization, Thoracoscopy with pleural mass biopsy was done and right-side chest tube was inserted (Fig. 2).

**Fig. 3:**
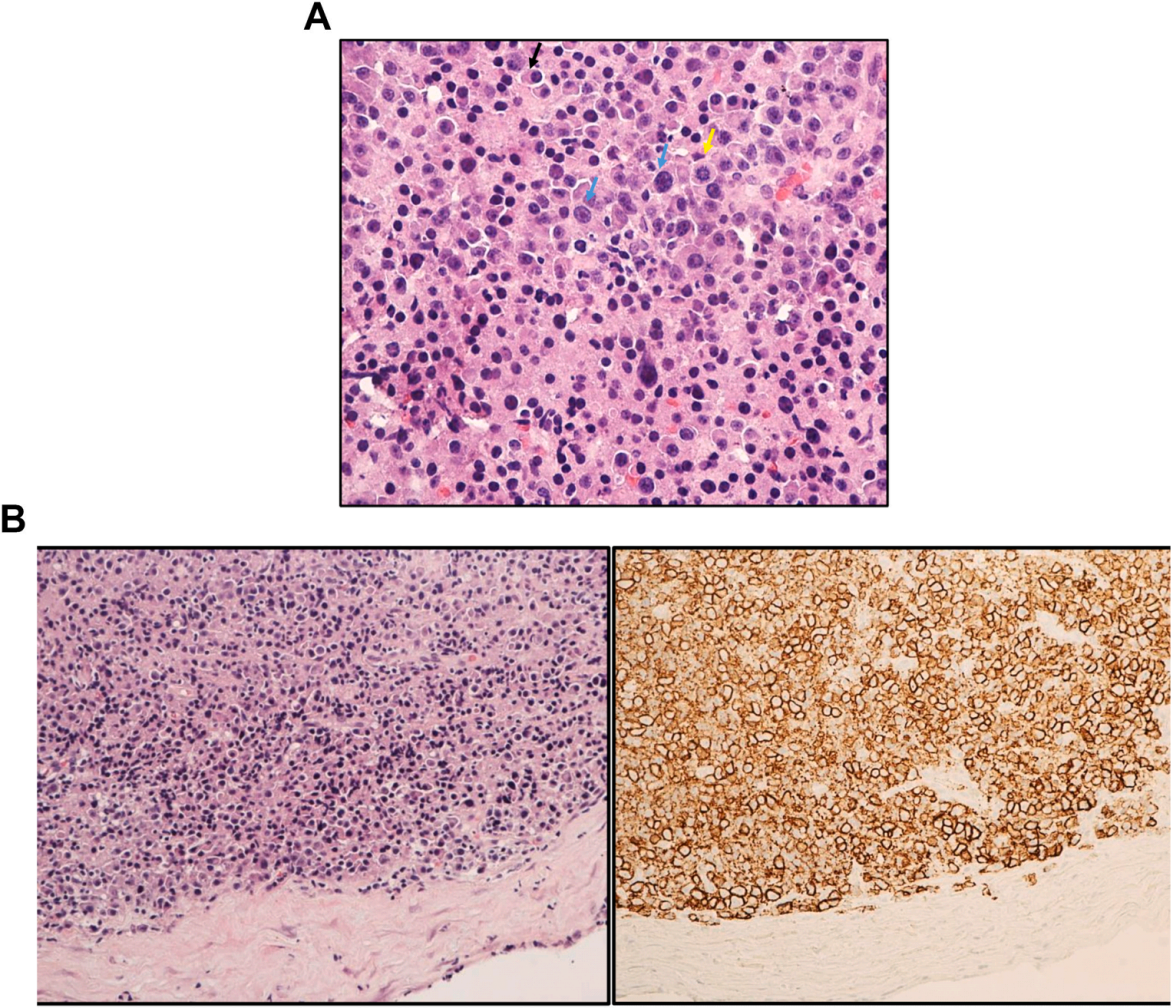
(A and B) section shows diffuse plasma cell proliferation composed of mixture of mature and immature plasma cells. Mature plasma cells have eccentric nuclei, clumped clock face chromatin pattern, and perinuclear hof (black arrows). Immature cells are variable in size with higher N:C ratio, bizarre nuclei, multinucleation and macronucleoli (blue arrows). Mitotic activity is present (yellow arrow). (H&E stain, × 40.

Biopsy confirmed the diagnosis of extramedullary plasmacytomas in the setting of multiple myeloma relapse (Fig. 3A and B).

The patient was started on pomalidomide, bortezomib, and dexamethasone with regimen of pomalidomide 4 mg by mouth (days 1–14), bortezomib 1.3 mg/m2 (days 1 and 8), and dexamethasone 20 mg by mouth (days 1, 8, and 15) every 21 days of a chemotherapy cycle. Current cycle: 14 day 8. Last received: 9/6/22. -William Scheer, PharmD.

**Fig. 4:**
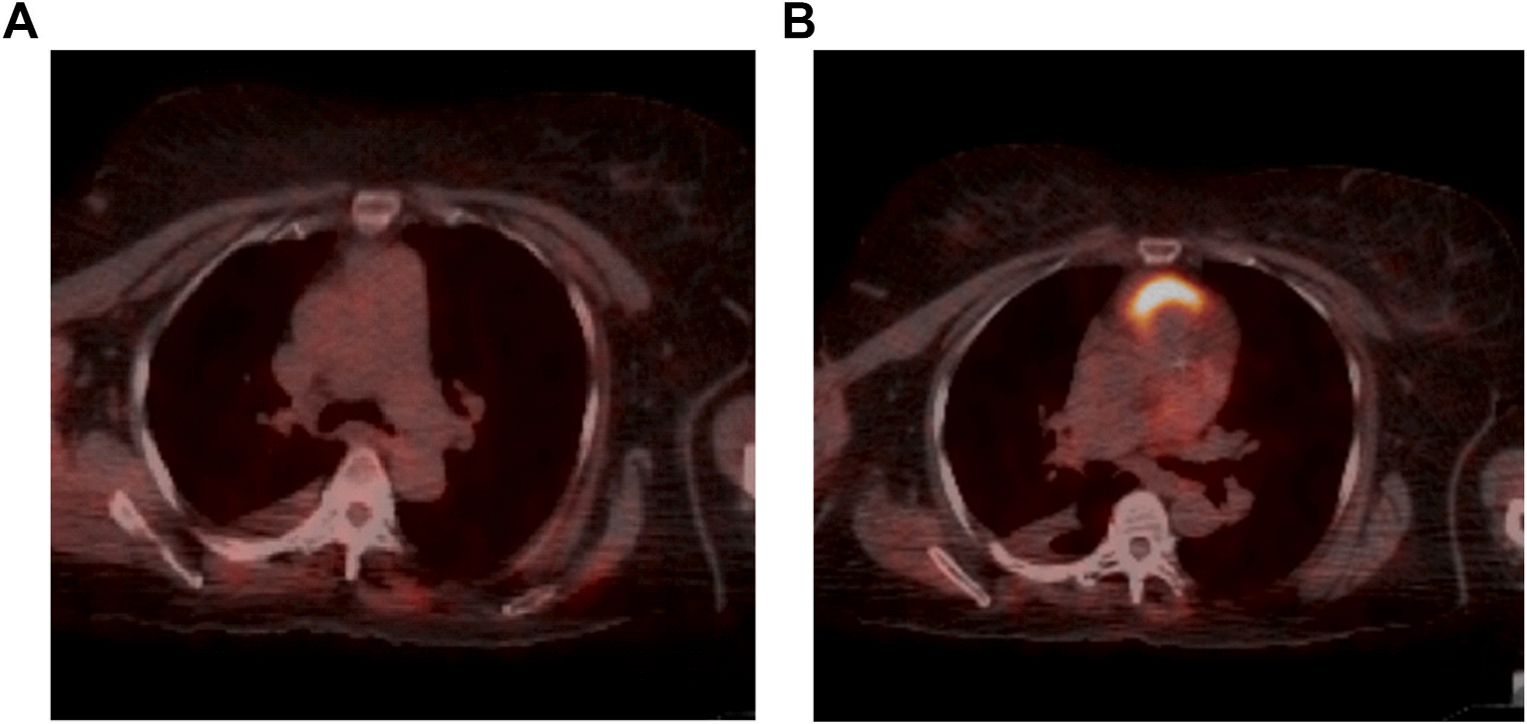
(A and B) Axial images of PET/CT scan which shows significant reduction of the PET activity pleural based masses.

On the fourth day, she was discharged home to be followed up in the outpatient clinic. A repeat PET scan after 6 months and after about 7 cycles of VPD therapy showed Interval diminution in PET activity in multiple bony foci, pleural based areas, left lung, cervical lymph node and right lateral chest wall consistent with a positive response to therapy (Fig. 4A and B). Then the patient maintained on the therapy.

## 3 Discussion

A plasmacytoma is a rare plasma cell neoplasm that develops within the bones or soft tissues structures and is considered to be an intermediate phase between MGUS and multiple myeloma (MM) [1]. Plasmacytomas typically present as a localized disease involving the bone or extramedullary sites, with no evidence of systemic involvement. A solitary plasmacytoma of bone (SPB) arises from the plasma cells of bone marrow and is typically seen in the bones of the axial skeleton, such as the vertebrae and skull. On the other hand, a solitary extramedullary or extraosseous plasmacytoma (SEP) develops from the plasma cells of mucosal surfaces and is frequently located in the upper aero-digestive tract, including the nasal cavity, sinuses, nasopharynx, and larynx [2].

However, it is important to note that extramedullary or extraosseous plasmacytomas can also develop as a part of systemic multiple myeloma (MM), which is characterized by the presence of ≥10% clonal plasma cells in the bone marrow [3]. Extramedullary disease may be an initial manifestation of MM or may herald the relapse of previously treated systemic disease. Extramedullary plasmacytoma during a relapse of MM should not be confused with a solitary extramedullary plasmacytoma (SEP), as both of these are considered to be two entirely different clinical entities [4,5]. Almost 15%–30% of patients may develop extramedullary involvement during the course of MM. Although rare, thoracic involvement has been reported in patients with multiple myeloma in the form of a lung mass, diffuse reticulonodular infiltration, multiple pulmonary nodules, lymphadenopathy and mediastinal mass, tracheobronchial infiltration, and nodular pleural thickening with pleural effusion [6]. Pleural plasmacytomas are extremely rare and account for around 3–6% of extramedullary disease in MM patients [4].

Pleural effusion or diffuse pulmonary infiltration by plasma cells is rare and usually occurs in advanced disease. Moreover, the development of pleural effusion in patients with MM is seldom a direct consequence of the myeloma itself, but is usually the result of a concurrent disease process, such as pneumonia, pulmonary embolism, or heart failure. Malignant myelomatous pleural effusion caused by the infiltration of pleural fluid by malignant plasma cells is seen in less than 1% of cases and may develop due to the extension of plasmacytomas of the chest wall, invasion from adjacent skeletal lesions, or lymphatic obstruction secondary to lymph node infiltration. It may also develop due to the direct implantation of tumor nodules on the pleura, i.e., pleural plasmacytomas [7,8].

Computed tomography (CT) and magnetic resonance imaging (MRI) may be useful in assessing the local extent and severity of extramedullary plasmacytomas. The high soft-tissue contrast and multiplanar directions confer an advantage to MRI. However, the detection of new disease activity in case of relapsed multiple myeloma (MM) using MRI or whole-body skeletal survey may be difficult. Fluorodeoxyglucose-positron emission tomography/computed tomography (FDG-PET/CT) allows the examination of the whole body in a single and faster study, and can help identify active disease in patients with relapsed or refractory MM by directly targeting the specific cellular properties of neoplastic plasma cells. This imaging technique has been found to offer superior detection of extramedullary disease, while providing important prognostic information [[9], [10], [11]]. Indeterminate pulmonary nodules or pleural lesions can be diagnosed by performing a transbronchial biopsy, CT-guided needle biopsy or a surgical biopsy through medical thoracoscopy, open thoracotomy or video-assisted thoracoscopic surgery (VATS). Medical thoracoscopy as we used in our case has a greater accuracy and is less invasive, and thus, has increasingly been used for this purpose [12]. An extramedullary plasmacytoma typically presents as a homogenous infiltrate of monoclonal plasma cells on histology. Characteristic immunohistochemical findings include the expression of CD138 and/or CD38. Following the diagnosis of plasmacytoma, complete blood count (CBC), serum calcium, renal function tests, serum protein electrophoresis (SPEP), urine protein electrophoresis (UPEP), skeletal survey, and bone marrow biopsy are performed to confirm or rule out the presence of multiple myeloma [9].

The treatment of solitary extramedullary plasmacytomas (SEPs), including pleural plasmacytomas, is with radiation, as these tumors are highly radiosensitive, and local control can be achieved in 80–100% of patients with a 10-year disease-free survival rate of 50–65% [12]. However, the treatment of extramedullary plasmacytomas in the setting of multiple myeloma (MM) relapse is rather challenging. A standard therapeutic guideline is not currently available and an individualized, multimodal approach using different strategies, such as advanced radiotherapy techniques, immunomodulatory agents (e.g., thalidomide, pomalidomide, and lenalidomide), and proteasome inhibitors (e.g., bortezomib, carfilzomib), is generally attempted. At the time of relapse, there is no clear rationale to prefer one drug class over another, and the therapeutic approach should take into account the previous lines of treatment as well as the duration of response in that patient [4,6,13,14]. The presence of extramedullary plasmacytomas in patients with multiple myeloma (MM) is associated with an adverse prognosis. The prognosis is even worse in patients who develop extramedullary disease at the time of relapse (median overall survival <6 months). Therefore, early detection and initiation of treatment are of utmost importance [7].

Our patient who remained in remission for approximately 9 years developed a solitary plasmacytoma of bone, and then almost one and a half years later, relapsed with multiple osteolytic lesions as well as multiple, biopsy-confirmed pleural plasmacytomas. Pleural involvement in patients with MM is rare and according to a recent literature review, less than 10 cases of pleural plasmacytomas have so far been reported in the literature [4]. Hence, this case also highlights the importance of being mindful of this rare diagnosis, as early recognition of this condition may have important implications for the management and prognosis of the patient. Further research is warranted to gain more knowledge regarding the exact pathogenesis of extramedullary recurrence in MM patients and to establish definitive treatment guidelines.

## 4 Conclusion

Extramedullary plasmacytoma involving the pleura is rare in patients with multiple myeloma (MM), and even more so in the setting of relapsed MM. However, it portends a poor prognosis and is associated with low progression-free and overall survival rates, which highlights the importance of swift diagnosis and treatment. Therefore, it is important for physicians to be aware of this rare condition, even in MM patients who are in complete remission. Fluorodeoxyglucose-positron emission tomography/computed tomography (FDG-PET/CT) is an effective tool in the diagnosis of pleural plasmacytomas and the diagnosis can be confirmed via biopsy. An individualized, multimodal approach, involving a combination of systemic and radiation therapy, may be used.

## Declaration of competing interest

All authors whose names are listed immediately below certify that they have no conflicts of interest.
